# Tie-2 Cre-Mediated Deficiency of Extracellular Signal-Regulated Kinase 2 Potentiates Experimental Bronchopulmonary Dysplasia-Associated Pulmonary Hypertension in Neonatal Mice

**DOI:** 10.3390/ijms21072408

**Published:** 2020-03-31

**Authors:** Renuka T. Menon, Amrit Kumar Shrestha, Roberto Barrios, Corey Reynolds, Binoy Shivanna

**Affiliations:** 1 Section of Neonatology, Department of Pediatrics, Baylor College of Medicine, Houston, TX 77030, USA; Renuka.Menon@bcm.edu (R.T.M.); Amrit.Shrestha@bcm.edu (A.K.S.); 2 Department of Pathology and Genomic Medicine, Houston Methodist Hospital, Houston, TX 77030, USA; rbarrios@houstonmethodist.org; 3 Mouse Phenotyping Core, Baylor College of Medicine, Houston, TX 77030, USA; reynolds.corey@att.net

**Keywords:** extracellular signal-regulated kinase 2, HPMECs, hyperoxia, bronchopulmonary dysplasia, pulmonary hypertension

## Abstract

Bronchopulmonary dysplasia (BPD)-associated pulmonary hypertension (PH) is a significant lung morbidity of infants, and disrupted lung angiogenesis is a hallmark of this disease. We observed that extracellular signal-regulated kinases (ERK) 1/2 support angiogenesis in vitro, and hyperoxia activates ERK1/2 in fetal human pulmonary microvascular endothelial cells (HPMECs) and in neonatal murine lungs; however, their role in experimental BPD and PH is unknown. Therefore, we hypothesized that *Tie2* Cre-mediated deficiency of *ERK2* in the endothelial cells of neonatal murine lungs would potentiate hyperoxia-induced BPD and PH. We initially determined the role of *ERK2* in in vitro angiogenesis using fetal HPMECs. To disrupt endothelial *ERK2* signaling in the lungs, we decreased *ERK2* expression by breeding *ERK2*^flox/flox^ mice with *Tie*-Cre mice. One-day-old endothelial *ERK2*-sufficient (*eERK2*^+/+^) or –deficient (*eERK2*^+/−^) mice were exposed to normoxia or hyperoxia (FiO_2_ 70%) for 14 d. We then performed lung morphometry, gene and protein expression studies, and echocardiography to determine the extent of inflammation, oxidative stress, and development of lungs and PH. The knockdown of *ERK2* in HPMECs decreased in vitro angiogenesis. Hyperoxia increased lung inflammation and oxidative stress, decreased lung angiogenesis and alveolarization, and induced PH in neonatal mice; however, these effects were augmented in the presence of *Tie2*-Cre mediated endothelial *ERK2* deficiency. Therefore, we conclude that endothelial *ERK2* signaling is necessary to mitigate hyperoxia-induced experimental BPD and PH in neonatal mice. Our results indicate that endothelial ERK2 is a potential therapeutic target for the management of BPD and PH in infants.

## 1. Introduction

Bronchopulmonary dysplasia (BPD) is a chronic lung disease of infants that results from interrupted lung development [1]. Despite continued improvements in the respiratory care management of preterm infants, the incidence of BPD continues to be high in extremely preterm infants [2]. There are no curative therapies for this disease, and the costs required to take care of an infant with BPD is twice that required to care for an infant without BPD [3]. Furthermore, pulmonary hypertension (PH) is a common complication of BPD [4], and the occurrence of this complication frequently increases short- and long-term mortality and morbidity in BPD infants [4,5,6,7,8]. Therefore, there is a need for improved therapies to prevent and/or treat BPD-associated PH. 

Interrupted lung development or alveolar and pulmonary vascular simplification is a characteristic pathologic finding of BPD [9,10]. Lung blood vessels are pivotal for healthy lungs. Lung angiogenesis promotes lung development, and disrupted angiogenesis can interrupt alveolarization in the developing lungs [11]. Therefore, understanding the molecular mechanisms that promote the development and function of the lung blood vessels is important to mitigate this human disease. To this end, the vascular endothelial growth factor (VEGF) and nitric oxide (NO) signaling pathways have been extensively investigated, and they have been shown to be necessary for lung development in health and disease in neonatal animals [12,13,14,15,16]. VEGF has been consistently shown to attenuate experimental BPD and PH via the endothelial nitric oxide synthase pathway [17,18,19]. However, these beneficial effects were not reproduced in humans [20,21]. Recent evidence suggests that a combination therapies such as inhaled NO combined with vitamin A can decrease the incidence of BPD better than NO therapy alone [22]. Thus, there is a need to identify additional druggable molecular targets that can complement inhaled NO therapy to promote the development and function of the lung vascular system. Our study indicates that the mitogen-activated protein (MAP) kinase, extracellular signal-regulated kinase (ERK) 2, is one such druggable target.

MAP kinases are downstream effectors of several growth factors that are involved in the complex and coordinated process of lung development [23]. Among the MAP kinases, ERK1/2 primarily mediate the proliferation and differentiation of many cell types, whereas c-Jun NH_2_-terminal kinases and p38 kinase mainly induce cell apoptosis [24]. ERK1/2 are active during the development of many organisms [25,26] and regulate morphogenesis in several organs, including the lungs [27,28,29]. Additionally, we demonstrated that ERK1/2 is required for angiogenesis in vitro, and hyperoxia exposure activates ERK1/2 in fetal human lung endothelial cells and in neonatal murine lungs [30]. We also observed that hyperoxia activates ERK2 more than ERK1 in human lung endothelial cells [30]. However, whether ERK2 contributes to or mitigates experimental BPD-associated PH is unknown. Furthermore, lung endothelial cells play a crucial role in normal lung development and the pathogenesis of BPD-associated PH. Therefore, this study aimed to decipher the role of endothelial *ERK2* in experimental BPD-associated PH.

Oxygen therapy is necessary to correct hypoxemia in preterm infants with respiratory failure; however, excessive oxygen exposure or hyperoxia contributes to lung injury and BPD pathogenesis. The phenotype of hyperoxia-induced lung injury in neonatal mice is identical to that of human BPD-associated PH [31,32,33,34]. Therefore, we used this model to elucidate the role of endothelial *ERK2* in experimental BPD-associated PH. Specifically, we tested the hypothesis that a *Tie2* Cre-mediated deficiency of endothelial *ERK2* in neonatal murine lungs will potentiate hyperoxia-induced BPD and PH. To increase the translational potential of our study, we also used fetal human pulmonary microvascular endothelial cells (HPMECs) to investigate the role of *ERK2* in the angiogenesis of developing human lungs. 

## 2. Results

### 2.1. ERK2 Is Required for HPMEC Tubule Formation

**Figure 1:**
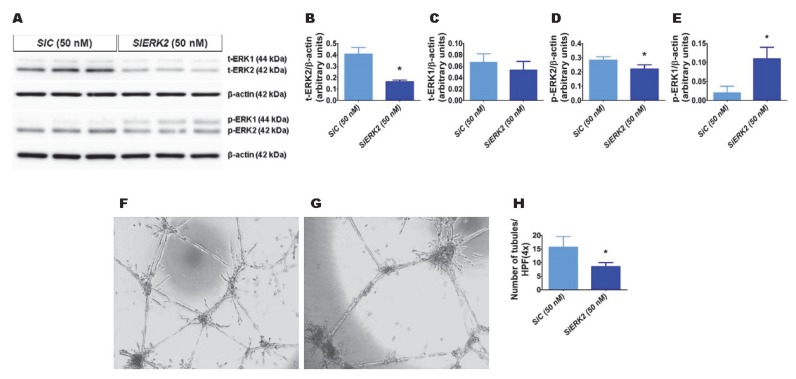
The effects of *ERK2* knockdown on human pulmonary microvascular endothelial cell (HPMEC) tubule formation. Control- or *ERK2*-siRNA-transfected HPMECs were harvested for protein expression and tubule formation assays. (**A**) The determination of total (t)- and phosphorylated (p)-extracellular signal-regulated kinase (ERK)2 and ERK1 protein levels in control (*SiC*)- and *ERK2* (*SiERK2)*-siRNA-transfected cells by immunoblotting. (**B**–**E**) Quantification and normalization of t-ERK2 (**B**), t-ERK1 (**C**), p-ERK2 (**D**), and p-ERK1 (**E**) band intensities to those of β-actin. (**F**–**H**) Representative photographs showing the tubule formation of cells transfected with control (**F**) and *ERK2* (**G**) siRNAs. (**H**) Quantification of tubule formation in control (*SiC*) and *ERK2* (*SiERK2*) siRNA-transfected cells. Values are presented as mean ± SD (n = 3/group for the protein expression studies and *n* = 7–8/group for the tubule formation assay). Significant differences between control siRNA- and *ERK2* siRNA-transfected groups are indicated by *, *p* < 0.05 (*t*-test).

We recently observed that hyperoxia exposure activates ERK2 more than ERK1 in fetal human lung endothelial cells [30]. Therefore, we initially determined whether ERK2 promotes HPMEC tubule formation. We used *ERK2* siRNA to knockdown ERK2 in HPMECs. *ERK2* siRNA efficiently decreased total ERK2 protein expression by greater than 2-fold without affecting the levels of total ERK1 protein (Figure 1A–C). The phosphorylation of ERK2 decreased (Figure 1A,D), whereas that of ERK1 increased (Figure 1A,E) with the knockdown of ERK2. Furthermore, the knockdown of ERK2 decreased HPMEC tubule formation (*SiEKR2*, 8.5 ± 1.5 vs. *SiC*, 15.7 ± 3.9; *p* < 0.001 (Figure 1F–H)), indicating that *ERK2* signaling is required for human lung endothelial cell tubule formation.

### 2.2. Lung ERK2 Expression Is Decreased in Endothelial ERK2-Deficient (eERK2^+/−^) Mice

**Figure 2:**
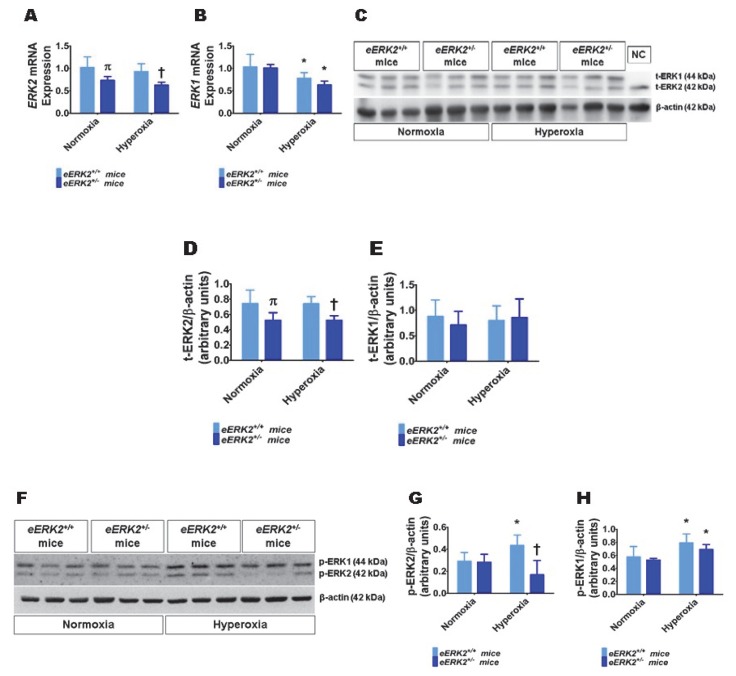
ERK1 and ERK2 mRNA and protein expression in the lungs of endothelial *ERK2*-sufficient (*eERK2*^+/+^) or -deficient (*eERK2*^+/−^) mice. One-day-old *eERK2*^+/+^ or *eERK2*^+/−^ mice were exposed to either 21% O_2_ (normoxia) or 70% O_2_ (hyperoxia) for one week, after which the lungs were harvested for gene and protein expression studies. (**A**,**B**) RT-PCR analysis-based determination of *ERK2* (**A**) and *ERK1* (**B**) mRNA levels. (**C**) Determination of total (t)-ERK1 and -ERK2 protein levels by immunoblotting. NC: negative control (lung sample from global *ERK1* knockout mice). (**D**,**E**) Quantification and normalization of t-ERK2 (**D**) and t-ERK1 (**E**) band intensities to those of β-actin. (**F**) Determination of phosphorylated (p)-ERK1 and -ERK2 protein levels by immunoblotting. (**G**,**H**) Quantification and normalization of *p*-ERK2 (**G**) and p-ERK1 (**H**) band intensities to those of β-actin. Values are presented as mean ± SD (*n* = 5–6/genotype/exposure). Significant differences between *eERK2*^+/+^ and *eERK2*^+/−^ mice in normoxic and hyperoxic conditions are indicated by π, *p* < 0.05 and †, *p* < 0.05, respectively. Significant differences between normoxia and hyperoxia groups are indicated by *, *p* < 0.05 (ANOVA).

Having observed that *ERK2* is essential for human lung endothelial cell tubule formation and that hyperoxia exposure increases ERK2 activation in human lung endothelial cells, we next investigated if endothelial cell *ERK2* signaling plays a mechanistic role in the pathogenesis of hyperoxia-induced lung injury in neonatal mice. To decrease *ERK2* expression in lung endothelial cells, we mated *Tie2*-Cre mice with *ERK2*-floxed mice and determined the expression of ERK1 and ERK2 mRNA and protein in the lungs of the offspring. ERK2 mRNA (Figure 2A) and protein levels (Figure 2C,D) were significantly decreased in the lungs of *eERK2*^+/−^ mice compared with their wild-type (WT) littermates. Importantly, ERK1 mRNA (Figure 2B) and protein levels (Figure 2C,E) were similar in the lungs of *eERK2*^+/−^ mice and their WT littermates, indicating that there was no compensatory overexpression of ERK1 in our experimental conditions. We also determined if the ERK1 and 2 activities were altered in these transgenic mice. Consistent with our in vitro cell experiments, the activity of ERK2, as determined by quantifying its phosphorylated protein levels, was decreased in *eERK2*^+/−^ mice exposed to normoxia or hyperoxia (Figure 2F,G). However, unlike the results from our in vitro experiments, the phosphorylated levels of ERK1 protein were comparable between e*ERK2^+/+^* and *eERK2*^+/−^ mice in either normoxia or hyperoxic conditions (Figure 2F,H). Additionally, our results indicate that hyperoxia exposure increases ERK1 and ERK2 activation in wild-type littermates (Figure 2F–H).

### 2.3. Endothelial ERK2 Deficiency Potentiates Hyperoxia-Induced Alveolar Simplification

**Figure 3:**
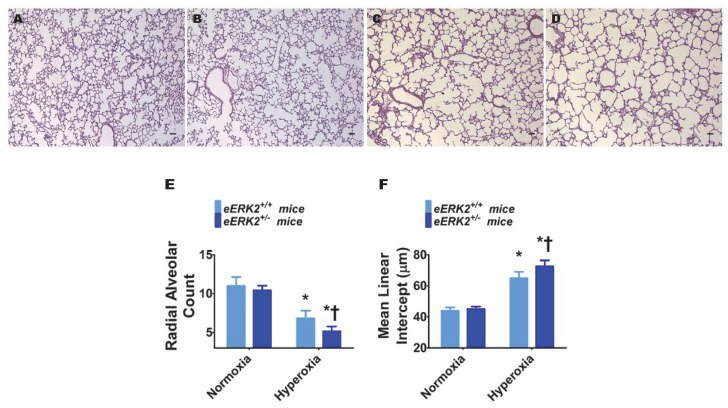
Lung alveolarization in endothelial *ERK2*-sufficient (*eERK2*^+/+^) or -deficient (*eERK2*^+/−^) mice exposed to hyperoxia. One-day-old *eERK2*^+/+^ or *eERK2*^+/−^ mice were exposed to either 21% O_2_ (normoxia) or 70% O_2_ (hyperoxia) for two weeks, after which the lungs were harvested for lung morphometry. (**A**–**D**) Representative hematoxylin and eosin-stained lung sections from *eERK2*^+/+^ (**A**,**C**) and *eERK2*^+/−^ (**B**,**D**) mice exposed to normoxia (**A**,**B**) or hyperoxia (**C**,**D**). Scale bar = 100 µm. (**E**,**F**) Alveolarization was quantified by determining radial alveolar counts (RAC) (**E**) and mean linear intercepts (MLI) (**F**). Values are presented as the mean ± SD (*n* = 4–5/genotype/exposure). Significant differences between *eERK2*^+/+^ and *eERK2*^+/−^ mice in hyperoxic conditions are indicated by †, *p* < 0.05. Significant differences between normoxia and hyperoxia groups are indicated by *, *p* < 0.05 (ANOVA).

Interrupted lung development that is characterized by fewer and larger alveoli, i.e., alveolar simplification, is a histopathologic hallmark of experimental BPD. Therefore, we next determined the impact of endothelial *ERK2* signaling on lung development by quantifying radial alveolar counts (RAC) and mean linear intercepts (MLI). Hyperoxia exposure decreased RAC (Figure 3A,C,E) while increasing the MLI (Figure 3A,C,F), suggesting that there were fewer and larger alveoli, respectively, in hyperoxia-exposed mice. These disruptive effects of hyperoxia on lung development were augmented in mice with deficient endothelial *ERK2* signaling (Figure 3B–F). Our observations indicate that endothelial *ERK2* is essential to mitigate hyperoxia-induced alveolar simplification.

### 2.4. Endothelial ERK2 Deficiency Potentiates Hyperoxia-Induced Pulmonary Vascular Simplification

**Figure 4:**
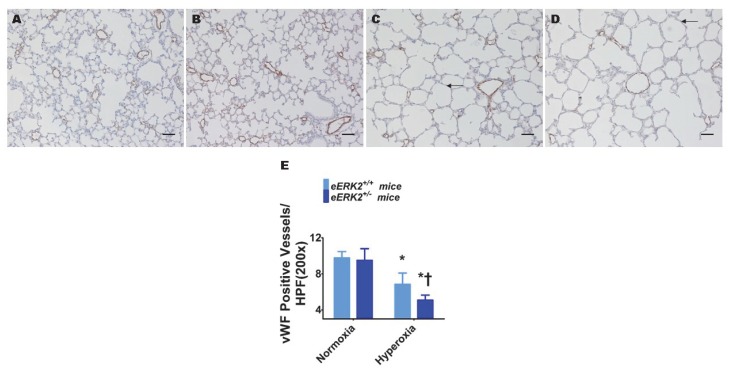
Lung vascularization in endothelial *ERK2*-sufficient (*eERK2*^+/+^) or -deficient (*eERK2*^+/−^) mice exposed to hyperoxia. One-day-old *eERK2*^+/+^ or *eERK2*^+/−^ mice were exposed to either 21% O_2_ (normoxia) or 70% O_2_ (hyperoxia) for two weeks, after which the lungs were harvested for lung vascular morphometry. (**A**–**D**) Representative von Willebrand factor (vWF)-stained lung sections from *eERK2*^+/+^ (**A**,**C**) and *eERK2*^+/−^ (**B**,**D**) mice exposed to normoxia (**A**,**B**) or hyperoxia (**C**,**D**). Scale bar = 100 µm. (**E**) Quantitative analysis of vWF-stained lung blood vessels per high power field (HPF). Values are presented as the mean ± SD (*n* = 4–5/genotype/exposure). Significant differences between *eERK2*^+/+^ and *eERK2*^+/−^ mice in hyperoxic conditions are indicated by †, *p* < 0.05. Significant differences between normoxia and hyperoxia groups are indicated by *, *p* < 0.05 (ANOVA).

Pulmonary vascular simplification, i.e., decreased and dysmorphic lung vasculature, is also a characteristic feature of experimental BPD. Hyperoxia exposure decreased lung vascularization as determined by the reduced number of von Willebrand factor (vWF)-stained lung blood vessels in hyperoxia-exposed mice (Figure 4A,C,E). Consistent with its effects on alveolar simplification, endothelial *ERK2* deficiency potentiated the effects of hyperoxia on pulmonary vascular simplification (Figure 4B–E), indicating that endothelial *ERK2* is essential to protect the lung vasculature against hyperoxic injury in neonatal mice.

### 2.5. Endothelial ERK2 Deficiency Potentiates Hyperoxia-Induced PH

**Figure 5:**
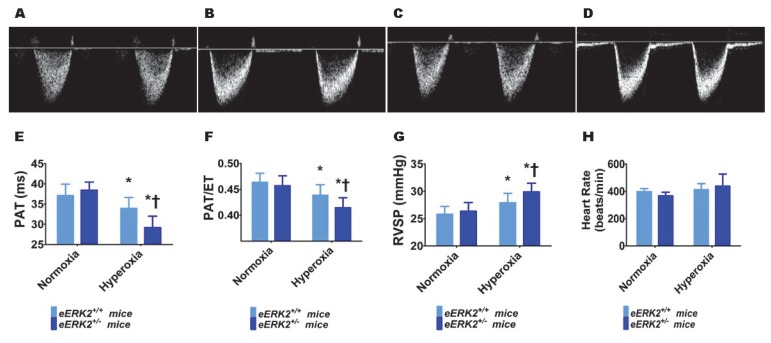
Indices of PH in endothelial *ERK2*-sufficient (*eERK2*^+/+^) or –deficient (*eERK2*^+/−^) mice exposed to hyperoxia. One-day-old *eERK2*^+/+^ or *eERK2*^+/−^ mice were exposed to either 21% O_2_ (normoxia) or 70% O_2_ (hyperoxia) for two weeks, after which high-resolution echocardiography studies were performed to estimate pulmonary hypertension (PH) in the experimental animals. (**A**–**D**) Representative PWD Echo recordings of pulmonary artery blood flow obtained from *eERK2*^+/+^ (**A**,**C**) and *eERK2*^+/−^ (**B**,**D**) mice exposed to normoxia (**A**,**B**) or neonatal hyperoxia (**C**,**D**). (**E**–**H**) Pulmonary acceleration time (PAT) (**E**), PAT/ejection time (ET) ratio (**F**), right ventricular systolic pressure (RVSP) (**G**), and heart rate (**H**) were estimated from the PWD Echo recordings of the pulmonary artery blood flow. Values are presented as mean ± SD (*n* = 4–5/genotype/exposure). Significant differences between *eERK2*^+/+^ and *eERK2*^+/−^ mice in hyperoxic conditions are indicated by †, *p* < 0.05. Significant differences between normoxia and hyperoxia groups are indicated by *, *p* < 0.05 (ANOVA).

Furthermore, we performed transthoracic echocardiography (Echo) studies on PND14 in our experimental animals to determine the impact of hyperoxia and endothelial *ERK2* signaling on PH. The heart rate was comparable in all of our experimental animals (Figure 5H). Hyperoxia exposure decreased the right ventricle (RV) systolic time intervals, pulmonary acceleration time (PAT) (Figure 5A,C,E) and the PAT/ejection time (ET) ratio (Figure 5A,C,F), and increased the estimated right ventricular systolic pressure (RVSP) (Figure 5G), indicating that hyperoxia induced PH in our experimental animals. However, endothelial *ERK2* deficiency potentiated hyperoxia-induced PH (Figure 5A–G), reiterating the protective effects of endothelial *ERK2* on lung vascular health in neonatal mice.

### 2.6. Endothelial ERK2 Deficiency Potentiates Hyperoxia-Induced Lung Inflammation

**Figure 6:**
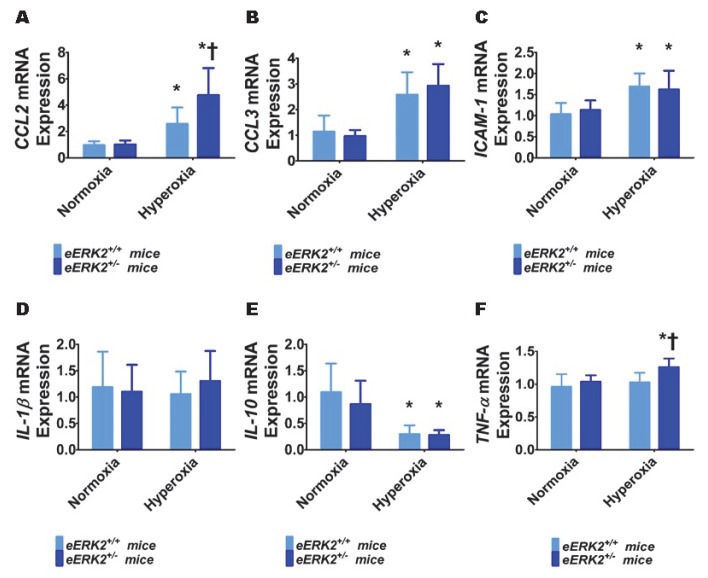
Lung inflammation in endothelial *ERK2*-sufficient (*eERK2*^+/+^) or –deficient (*eERK2*^+/−^) mice exposed to hyperoxia. One-day-old *eERK2*^+/+^ or *eERK2*^+/−^ mice were exposed to either 21% O_2_ (normoxia) or 70% O_2_ (hyperoxia) for one week, following which the lung tissues were harvested for gene expression studies. (**A**–**F**) RT-PCR analyses-based determination of *CCL2* (**A**), *CCL3* (**B**), *ICAM-1* (**C**), *IL-1β* (**D**), *IL-10* (**E**), and *TNF-α* (**F**) mRNA levels. Values are presented as mean ± SD (*n* = 5–6/genotype/exposure). Significant differences between *eERK2*^+/+^ and *eERK2*^+/−^ mice in hyperoxic conditions are indicated by †, *p* < 0.05. Significant differences between normoxia and hyperoxia groups are indicated by *, *p* < 0.05 (ANOVA).

To determine the mechanisms through which endothelial *ERK2* deficiency exerts its detrimental effects, we evaluated the extent of lung inflammation in our experimental groups at PND7. Specifically, we quantified the generation of pro-inflammatory cytokines (*chemokine (C-C motif) ligand* (*CCL) 2*, *CCL3, intercellular adhesion molecule (ICAM)-1, interleukin (IL)-1β*, and *tumor necrosis factor (TNF)-α*) and an anti-inflammatory cytokine (*IL-10*) in lung tissues by real-time RT-PCR. Hyperoxia exposure for 7 d increased the mRNA levels of pro-inflammatory cytokines, *CCL2* (Figure 6A), *CCL3* (Figure 6B), and *ICAM-1*(Figure 6C), and decreased the mRNA level of the anti-inflammatory cytokine, *IL-10* (Figure 6E). Hyperoxia exposure for 7 d did not independently affect the mRNA levels of the pro-inflammatory cytokines, *IL-1β* (Figure 6D) and *TNF-α* (Figure 6F) in our experimental animals. Among these cytokines, we observed that endothelial *ERK2* deficiency preferentially affected the expression of *CCL2* and *TNF-α*. Endothelial *ERK2* deficiency potentiated the effects of hyperoxia on *CCL2* expression (Figure 6A). Importantly, endothelial *ERK2* deficiency independently increased the expression of *TNF-α* (Figure 6F) in hyperoxic conditions, indicating that increased lung inflammation may be partly responsible for the augmented hyperoxic lung injury in *eERK2*^+/−^ mice.

### 2.7. Endothelial ERK2 Deficiency Potentiates Hyperoxia-Induced Oxidative Stress

**Figure 7:**
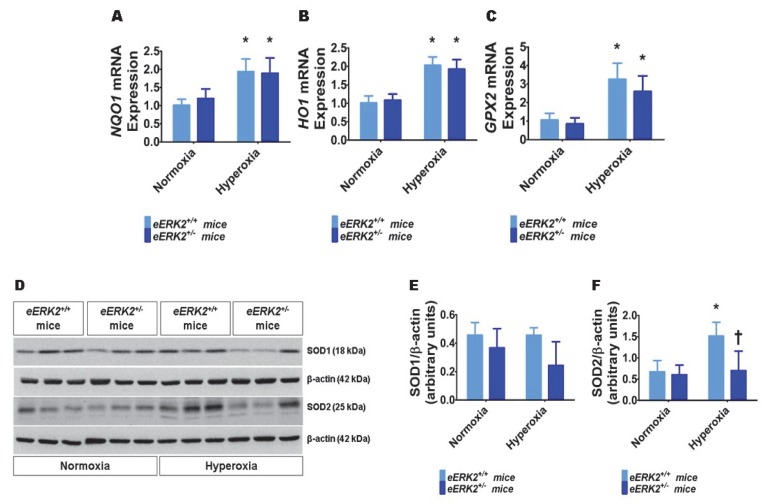
Lung oxidative stress in endothelial *ERK2*-sufficient (*eERK2*^+/+^) or –deficient (*eERK2*^+/−^) mice exposed to hyperoxia. One-day-old *eERK2*^+/+^ or *eERK2*^+/−^ mice were exposed to either 21% O_2_ (normoxia) or 70% O_2_ (hyperoxia) for one week, following which the lung tissues were harvested for gene expression studies. (**A**–**C**) RT-PCR analyses-based determination of *NQO1* (**A**), *HO1* (**B**), and *GPX2* (**C**) mRNA levels. (**D**) Determination of SOD1 and SOD2 protein levels by immunoblotting. (**E**,**F**) Quantification and normalization of SOD1 (**E**) and SOD2 (**F**) band intensities to those of β-actin. Values are presented as mean ± SD (*n* = 5–6/genotype/exposure). Significant differences between normoxia and hyperoxia groups are indicated by *, *p* < 0.05 (ANOVA).

Besides lung inflammation, oxidative stress is an important mediator of hyperoxic lung injury. Therefore, we finally determined the extent of lung oxidative stress in our experimental groups by quantifying the mRNA levels of the well-known anti-oxidant enzymes, *NAD(P)H quinone dehydrogenase 1* (*NQO1*), *heme oxygenase 1* (*HO1*), and *glutathione peroxidase 2* (*GPX2*) by real-time RT-PCR, and the protein levels of superoxide dismutase (SOD) 1 and SOD2 by immunoblotting. Hyperoxia increased the mRNA levels of *NQO1* (Figure 7A), *HO1* (Figure 7B), and *GPX2* (Figure 7C) in the lungs, suggesting that hyperoxia-induces oxidative stress and the neonatal mice attempt to alleviate this stress by increasing the generation of the anti-oxidant enzymes. However, the mRNA levels of these anti-oxidant enzymes were comparable between *eERK2*^+/+^ and *eERK2*^+/−^ mice (Figure 7A–C). Furthermore, hyperoxia increased SOD2 but not SOD1 protein levels in the lungs (Figure 7D–F) in our hyperoxia model. Interestingly, the effect hyperoxia on SOD2 protein levels was significantly abrogated in *eERK2*^+/−^ mice (Figure 7D,F). These findings indicate that increased oxidative stress may also be partly responsible for the augmented hyperoxic lung injury in neonatal *eERK2*^+/−^ mice.

## 3. Discussion

In the present study, we investigated the effects of *ERK2* deficiency on human fetal lung endothelial cell tubule formation *in vitro*, as well as the effects of *Tie2*-Cre mediated endothelial *ERK2* deficiency on hyperoxic lung injury in neonatal mice *in vivo*. Our *in vitro* experiments indicate that *ERK2* deficiency disrupts human fetal lung endothelial cell tubule formation. Further, our *in vivo* experiments in neonatal mice demonstrate that *Tie2*-Cre mediated endothelial *ERK2* deficiency potentiates hyperoxia-induced alveolar and pulmonary vascular simplification, PH, and lung inflammation and oxidative stress.

Lung blood vessels orchestrate alveolarization during development. Disrupted lung angiogenesis inhibits alveolar development and leads to BPD. Furthermore, decreased growth, and altered vasoreactivity and extracellular matrix of the lung endothelial cells contribute to the pathogenesis of PH in BPD [11,35,36,37]. Therefore, understanding the mechanisms that promote the development and function of the lung vascular system is vital to prevent and treat BPD and PH in human infants. The majority of infants who develop BPD-PH are born preterm, as early as 22 weeks gestational age. Therefore, we used lung endothelial cells from fetuses of similar gestational age and stage of lung development to elucidate the mechanisms contributing to BPD-PH. Several studies [38,39], including ours [30], indicate that ERK1/2 is one of the many molecules that protect and promote vascular health of lungs and other organs. However, it is unclear if ERK1 or ERK2 or both of them are necessary for these beneficial effects. Our previous study showed that hyperoxia, a common insult used to induce experimental BPD and PH, activates ERK2 greater than ERK1 in fetal HPMECs [30]. So, we focused mainly on the role of endothelial *ERK2* in the pathogenesis of experimental BPD and PH in this investigation. Initially, we investigated if *ERK2* signaling is necessary for human fetal lung endothelial cell angiogenesis. Our findings demonstrate *ERK2* is necessary for angiogenesis during development. Several studies have demonstrated the combined proangiogenic effects of *ERK1* and *ERK2*; however, our study differs from these by demonstrating the proangiogenic effects of *ERK2* in isolation.

Based on the findings of our *in vitro* studies, we next used neonatal mice to investigate the role of endothelial *ERK2* in the pathogenesis of experimental BPD and PH. We used *Tie2* driven Cre recombinase to regulate *ERK2* expression in the lung endothelial cells. ERK2-deficiency increased ERK1 activity in our *in vitro* experiments, but not in our *in vivo* experiments. There are a few possibilities for these discrepant findings. For one, we achieved a significant knockdown of ERK2 in HPMECs, whereas the deficiency of ERK2 was modest in our endothelial-specific *ERK2*-deficient mice. Second, we used human lung endothelial cells to quantify ERK1 activity in our *in vitro* experiments, whereas we used the whole lung lysates to determine ERK1 activity in mice. The ERK1/2 enzymes are ubiquitously expressed in all cell types of lung tissue. Therefore, it is possible the ERK1 activity in non-endothelial cells may have contributed to the lack of differences in ERK1 activity between endothelial-specific *ERK2*-deficient mice and their wild-type littermates. We will address these possibilities in the future by using endothelial complete *ERK2* knockout mice and determining the ERK1 activity in their lung endothelial cells. It’s important to note that *Tie2* is also expressed in subsets of monocytes and macrophages in addition to endothelial cells. Therefore some of the phenotype seen in our model may reflect deficiency of the *ERK2* in these hematopoietic cells. However, *Tie2* is predominantly present in endothelial cells, and *Tie2*-Cre mice continue to be frequently used to elucidate the role of endothelial signaling in lung health and disease [40,41,42].

Hyperoxia impairs signaling pathways necessary for lung development and repair [43,44], disrupts alveolarization and pulmonary vascularization in preterm infants [45] and newborn mice [46], and causes BPD. Furthermore, our hyperoxia model is shown to recapitulate the short- and long-term phenotype of BPD infants with PH [33,34]. Consistent with this notion, our hyperoxia-exposed animals displayed alveolar and pulmonary vascular simplification. Importantly, these hyperoxia effects were augmented in endothelial *ERK2s-*deficient animals. These observations reinforce the importance of lung vascular health in lung development i.e., vascular hypothesis [11,47]. Furthermore, our findings emphasize the essential role of endothelial *ERK2* in promoting lung angiogenesis and mitigating developmental lung injury when exposed to an insult such as hyperoxia. Global *ERK1* knockout mice are viable [48], whereas global *ERK2* knockout in mice results in embryonic lethality [49], suggesting that ERK1 and ERK2 perform distinct roles in several tissues, including the vasculature. Recently, Ricard and colleagues demonstrated that loss of ERK2 in murine endothelial cells decreased arteriogenesis, whereas loss of endothelial ERK1 resulted in increased but poorly functional arteriogenesis [50]. Our findings reiterate this vasculoprotective role of ERK2. Evidence indicates that ERK1 and ERK2 exhibit functional redundancy and that global ERK quantity, but not isoform specificity, is a crucial determinant of ERK function and the resulting phenotype [51,52]. Therefore, the phenotype associated with our *in vivo* model may be due to the lack of an increase in ERK1 activity to compensate for the ERK2 deficiency. 

Consistent with our previous studies [30], hyperoxia exposure activated ERK1 and ERK2 proteins in wild-type littermates. Collectively, these observations indicate that the activities of ERK1/2 are increased as an adaptive response to hyperoxia exposure. Although the role(s) of ERK1/2 is well known in the field of endothelial lung biology, their role(s) in the pathogenesis of lung injury is open for debate. It’s unclear whether they promote or mitigate lung injury. For instance, Ahmad et al. [53] showed that ERK1/2 activation protects adult human lung pulmonary microvascular endothelial cells from hyperoxic injury. Similarly, Kim et al. [54] recently demonstrated that the formyl peptide receptor 2 agonist, WKYMVm hexapeptide, attenuates hyperoxic lung injury in neonatal mice via ERK1/2 activation. By contrast, Zhang et al. [55] have demonstrated that inhibition of ERK1/2 signaling mitigates hyperoxic injury in adult rodents. Likewise, Carnesecchi et al. [56] demonstrated that inhibition of oxidant injury-mediated ERK1/2 activation is one of the main mechanisms through which NADPH oxidase-1 attenuates hyperoxic lung injury in adult mice. ERK1/2 activation is also shown to augment hyperoxic lung injury in neonatal rodents, primarily by regulating proliferation and differentiation of fibroblasts [57,58]. Alveolar interstitial fibroblasts regulate lung development. Differentiation of alveolar interstitial fibroblast into lipofibroblast promotes lung development [59,60], while their differentiation into myofibroblast inhibits lung development [60]. Based on all these observations, it’s reasonable to justify that the biological response of ERK1/2 activation is dependent upon the cell and tissue types, magnitude and duration of ERK1/2 activation, and the interactions between ERK1/2 and other activated pathways. Our study indicates that deficiency of endothelial *ERK2* potentiates neonatal hyperoxic lung injury.

Echo is used increasingly to phenotype PH in mice due to the technical difficulty of obtaining accurate pulmonary arterial (PA) pressure by cardiac catheterization. Furthermore, Echo is feasible and reliable for determining PH in these experimental animals, and the RV systolic time intervals such as PAT and PAT/ET ratio estimated using high-frequency Echo closely correlate with the cardiac catheterization-based estimation of PA pressures [34,61,62]. Because increased PA pressures cause the pulmonary valve to close prematurely and decrease the PAT and PAT/ET ratio, our findings suggest that hyperoxia-induces a PH phenotype, and this phenotype is augmented by endothelial *ERK2* deficiency in neonatal mice. Interventions used to rescue the PH phenotype in adult rodents decrease ERK1/2 activation [63,64,65,66], suggesting that activation of these kinases promotes the development of PH. Similarly, Young et al. [67] demonstrated that inhibition of c-kit signaling mitigates hypoxia-induced PH in neonatal mice by downregulating activation of ERK1/2. Increased proliferation of pulmonary vascular smooth muscle cells and adventitial fibroblasts, leading to pulmonary vascular remodeling is a crucial pathogenic mechanism of PH. It’s possible that ERK1/2 signaling contributes to the pathogenesis of PH by promoting the proliferation of these cells that mediate pulmonary vascular remodeling. However, endothelial cell health and proliferation are pivotal to prevent and attenuate PH associated with BPD [11,47,68,69], and mitogens/growth factors such as ERK1/2 are important to promote these endothelial cell properties. Our findings support this concept, emphasizing the protective role of endothelial *ERK2* signaling in hyperoxia-induced PH. Importantly, our study differs from others because we have demonstrated a direct cause-effect relationship by endothelial- specific genetic manipulation rather than showing an indirect association between ERK1/2 activation and experimental PH. 

Persistent inflammation is a final common pathogenic mechanism of BPD in infants [70,71]. Consistent with these findings and the results of several pre-clinical studies of neonatal lung injury [70], hyperoxia increased the mRNA-expression levels of pro-inflammatory chemokines and cytokines and decreased the anti-inflammatory cytokine, *IL-10*, in our experimental model. The pro-inflammatory chemokines recruit and activate granulocytes, monocyte/macrophages, and T cells to secrete additional cytokines, leading to a vicious inflammatory cycle. Furthermore, they can activate and alter the phenotype of resident lung epithelial and endothelial cells from one that promotes lung development to that which augments lung inflammation [72,73]. Therefore, our findings indicate that increased lung inflammation is partly responsible for augmented hyperoxic lung injury in endothelial *ERK2* deficient-neonatal mice. The precise molecular mechanisms through which *ERK2* regulates inflammation in the developing lungs are unclear and warrant further investigation.

Oxidative stress is also a major pathogenic determinant of BPD. Hyperoxia-induced reactive oxygen species (ROS) generation contributes to chronic lung injury by altering signal transduction pathways and modifying the structure and function of the protein, lipids, and DNA [74,75]. However, it is difficult to quantify ROS in real-time as they are very unstable. Therefore, the majority of studies measure the expression of anti-oxidant enzymes to indirectly determine the extent of oxidative stress because these enzymes are up-regulated as an adaptive response when exposed to hyperoxia. Furthermore, the levels of these anti-oxidant enzymes also determine the extent of oxidative injury. For instance, increased expression of these enzymes confers protection against oxidant injury [76,77,78,79,80]. The decreased levels of the anti-oxidant enzyme, SOD2, in hyperoxia-exposed endothelial *ERK2-*deficient mice indicate that increased oxidative stress may also be a mechanism through which endothelial *ERK2* deficiency potentiates hyperoxic lung injury in our model. It’s important to point out that there is a weak correlation between mRNA/protein levels and total enzyme activity of SOD. Nevertheless, given the extent of decrease in SOD2 protein levels in endothelial-specific *ERK2*-deficient mice, we expect the corresponding enzyme activity also to be significantly reduced in these mice. The mechanisms through which ERK2 regulates SOD2 protein levels in hyperoxic conditions are unclear at present and warrant further investigation.

Our study has several strengths. One, we used human fetal lung endothelial cells to determine the role of *ERK2* in human lung angiogenesis, which increases the translational potential of our study. Two, we used a robust genetic approach to elucidate the role of endothelial *ERK2* in neonatal hyperoxic lung injury. Three, we used high-resolution Echo to determine the effects of endothelial *ERK2* deficiency on pulmonary vascular function. We also recognize that our study has a few limitations, which will be addressed in our future investigations. First, we did not examine the sex-specific effects of endothelial *ERK2* deficiency in neonatal hyperoxic lung injury. Second, we did not determine the impact of hyperoxia or endothelial *ERK2* deficiency on lung function. Finally, we did not investigate the precise molecular mechanisms through which endothelial *ERK2* deficiency potentiates neonatal hyperoxic lung injury. 

In summary, we demonstrate that *ERK2* signaling is necessary for HPMEC tubule formation and thereby, probably for human pulmonary vascular development. Furthermore, our in vivo studies show that *Tie2*-Cre mediated endothelial *ERK2* deficiency potentiates hyperoxia-induced experimental BPD and PH in neonatal mice. To the best of our knowledge, ours is the first study to elucidate the role of endothelial *ERK2* signaling in hyperoxia-induced experimental BPD and PH. Our findings signify that targeting endothelial *ERK2* signaling may benefit BPD infants who develop PH. 

## 4. Materials and Methods

### 4.1. In Vitro Experiments

#### 4.1.1. Cell Culture 

The HPMECs derived from the lungs of human fetus (18 weeks gestational age) were obtained from ScienCell research laboratories (San Diego, CA, USA; 3000) and grown in 95% air and 5% CO_2_ at 37 ºC according to the manufacturer’s recommendations. Briefly, the cells were grown in fibronectin coated plates containing basal endothelial cell medium supplemented with fetal bovine serum, antibiotics, and endothelial cell growth supplement in a humidifier containing 5% CO_2_ at 37 ºC. When the cell culture reached >90% confluence, they were subcultured with a split ratio of 1:3. Cells between passages 5–7 were used for all our experiments. 

#### 4.1.2. Transfection Experiments

The HPMECs were transfected for 24 h with either 50 nM control siRNA (Dharmacon, Lafayette, CO; d-001810) or 50 nM *ERK2* siRNA (Dharmacon; L-003555) using Lipofectamine RNAiMAX (Life Technologies, Grand Island, NY, USA; 13778075). The cells were then harvested to determine the effects of *ERK2* knockdown on ERK1/2 expression and *in vitro* angiogenesis.

#### 4.1.3. Western Blot Assays 

Cells were grown on six-well plates to 60% confluence and transfected with 50 nM control or *ERK2* siRNA for 24 h. Following these transfections, whole-cell protein extracts were obtained by using radioimmunoprecipitation assay lysis buffer system (Santa Cruz Biotechnologies, Santa Cruz, CA; sc-24948) and subjected to western blotting with the following antibodies: anti-β-actin (Santa Cruz Biotechnologies; sc-47778, dilution 1:1000), anti-total ERK1/2 (Cell Signaling, Danvers, MA; 4695, dilution 1:1000) and anti-phospho ERK1/2 (Cell Signaling; 9106, dilution 1:1000). The primary antibodies were detected by incubation with the appropriate horseradish peroxidase-conjugated secondary antibodies. The immunoreactive bands were detected by chemiluminescence method, and the band densities were quantified by Image lab 5.2.1 software (Bio-Rad Laboratories, Inc., Hercules, CA, USA) [81].

#### 4.1.4. Tubule Formation Assay

Tubule formation was determined by Matrigel assay as described before [82,83]. Briefly, HPMECs transfected with control or *ERK2* siRNA for 24 h were loaded on top of growth factor-reduced Matrigel (Corning, New York, NY, USA; 356230) and grown in 96-well microplates at a density of 2 × 10^4^ cells per well in 100 µL of complete ECM medium. The tubule formation was quantified following an incubation period of 18 h. 

#### 4.1.5. Statistical Analyses

The results were analyzed by GraphPad Prism 5 software. Data are expressed as mean ± SD. At least two separate experiments were performed for each measurement. The differential effects of *ERK2* siRNA transfection on ERK1/2 expression and tubule formation were determined by *t*-test. A *p* value of <0.05 was considered significant.

### 4.2. In Vivo Experiments

#### 4.2.1. Animals 

This study was approved and conducted in strict accordance with the federal guidelines for the humane care and use of laboratory animals by the Institutional Animal Care and Use Committee of Baylor College of Medicine. *Tie2*-Cre (B6.Cg-Tg(Tek-cre)1Ywa/J, 008863) and *ERK2*^flox/flox^ (B6.129-Mapk1tm1Gela/J, 019112) mice on a C57BL/6 background were obtained from The Jackson Laboratory (Bar Harbor, ME). Timed-pregnant mice raised in our animal facility were used for the experiments. To disrupt endothelial *ERK2* signaling in the lungs, we decreased endothelial *ERK2* expression by breeding *ERK2*^flox/flox^ mice with *Tie2*-Cre mice. The genotype of the mice was confirmed by performing polymerase chain reaction using primers as recommended by The Jackson Laboratory. The dams were fed standard mice food and water *ad libitum* and all the experimental animals were maintained in 12-h day/night cycles. 

#### 4.2.2. Hyperoxia Experiments

Endothelial *ERK2*-sufficient (*eERK2*^+/+^) or –deficient (*eERK2*^+/−^) mice were exposed to normoxia (FiO_2_ 21%) or hyperoxia (FiO_2_ 70%) through postnatal days (PNDs) 1–14. The dams were rotated between normoxia- and hyperoxia-exposed litters every 24 h during the experiment to prevent oxygen toxicity in the dams. Oxygen exposures were conducted in Plexiglas chambers and the animals were monitored as described previously [84].

#### 4.2.3. Tissue Preparation for Lung Morphometry Studies and Analyses of Lung Development

Experimental animals were euthanized at PND14, and their lungs were inflated and fixed via the trachea with 10% formalin at 25 cm H_2_O pressure for at least 10 min. Sections of the paraffin-embedded lungs were obtained for the analysis of lung alveolarization and vascularization, as described previously [84]. Alveolar growth was evaluated by quantifying radial alveolar counts (RAC) and mean linear intercepts (MLI), whereas pulmonary vessel density was determined based on immunohistochemical staining for von Willebrand factor (vWF) [34]. The number of vWF-stained blood vessels with a diameter of less than 150 µm was quantified to determine the pulmonary vascular density, as described previously [34]. 

#### 4.2.4. Lung Tissue Harvest for Gene and Protein Expression Studies

At PND7, the lungs from a subset of study animals were snap frozen in liquid nitrogen and stored at −80 °C for the subsequent isolation of total RNA and protein. Total lung RNA was isolated from the lungs and reverse transcribed to cDNA, as we have described before [85]. For total protein extraction, a mortar and pestle were used to homogenize the lung tissue in T-PER™ Tissue Protein Extraction Reagent (Thermo Fisher Scientific; 78510) with Halt™ Protease and Phosphatase Inhibitor Cocktail (Thermo Fisher Scientific; 78442). The homogenate was centrifuged at 10,000 g for 5 min at 4 °C. The supernatant, the protein lysate, was stored at −80 °C for subsequent studies.

#### 4.2.5. Real-Time RT-PCR Assays

Real-time quantitative RT-PCR analysis was performed using the TaqMan gene expression master mix (Grand Island, New York; 4369016) and the following TaqMan gene specific primers: *chemokine (C-C motif) ligand 2* (*CCL2*; Mm00441242_m1), *CCL3* (Mm00441259_g1), *ERK1* (Mm01973540_g1 ); *ERK2* (Mm00442479_m1 ), *heme oxygenase 1* (*HO1*; Mm00516005_m1), *intercellular adhesion molecule-1* (*ICAM-1*; Mm00516023_m1), *interleukin (IL)-1β* (*IL-1β*; Mm00434228_m1), *IL-10* (Mm01288386_m1), *NAD(P)H quinone dehydrogenase 1* (NQO1; Mm01253561_m1), *tumor necrosis factor-α* (*TNF-α*; Mm00443258_m1), *glutathione peroxidase 2* (*GPX2;* Mm00850074_g1), and *glyceraldehyde 3-phosphate dehydrogenase* (*GAPDH*; Mm99999915_g1). *GAPDH* was used as the reference gene, and the ΔΔC_t_ method was used to calculate the fold change in mRNA expression.

#### 4.2.6. Western Blot Assays

The protein lysates were separated by 10% SDS-polyacrylamide gel electrophoresis and transferred to polyvinylidene difluoride membranes. The membranes were incubated overnight at 4 °C with the following primary antibodies: anti-β-actin (Santa Cruz Biotechnologies; sc69879, dilution 1:5000), anti-total ERK1/2 (Cell Signaling, Danvers, MA, USA; 4695, dilution 1:1000), anti-phospho ERK1/2 (Cell Signaling; 9106, dilution 1:1000), anti-SOD1 (Santa Cruz Biotechnologies; sc-8637, dilution 1:1000), and anti-SOD2 (Santa Cruz Biotechnologies; sc-137254, dilution 1:1000). The immunoreactive bands were detected by the chemiluminescence method, as described in our in vitro experiments.

#### 4.2.7. Echocardiography

Echocardiography, using the VisualSonics Vevo 2100 machine and a 40 MHz linear transducer, was performed on PND14 to assess the indices of PH [34]. Pulsed-wave Doppler (PWD) recording of the pulmonary blood flow obtained at the level of the aortic valve in the parasternal right ventricular outflow view [61] was used to estimate pulmonary acceleration time (PAT) and right ventricle (RV) ejection time (ET). The RV systolic pressure (RVSP) was calculated using the regression formula RVSP = 64.5 − (83.5 × PAT/ET) [61]. 

#### 4.2.8. Statistical Analyses

The results were analyzed by the GraphPad Prism 5 software. Data are expressed as mean ± SD. The individual and interactive effects of *ERK2* gene expression and hyperoxia exposure on lung inflammation, alveolarization, pulmonary vascularization, and PH were assessed using two-way ANOVA. A *p* value of <0.05 was considered significant.

## Abbreviations

BPD	bronchopulmonary dysplasia
*CCL*	chemokine (C-C motif) ligand
Echo	echocardiography
ERK	extracellular signal-regulated kinases
*eERK2*^+/+^ mice	endothelial *ERK2*-sufficient mice
*eERK2*^+/−^ mice	endothelial *ERK2*-deficient mice
ET	ejection time
*GAPDH*	glyceraldehyde 3-phosphate dehydrogenase
*GPX2*	glutathione peroxidase 2
*ICAM-1*	intercellular adhesion molecule 1
*IL*	interleukin
HPMECs	human pulmonary microvascular endothelial cells
*HO1*	heme oxygenase 1
MAP kinase	mitogen-activated protein kinase
MLI	mean linear intercept
NO	nitric oxide
*NQO1*	NAD(P)H quinone dehydrogenase 1
PAT	pulmonary acceleration time
PH	pulmonary hypertension
PND	postnatal day
RAC	radial alveolar count
ROS	reactive oxygen species
RV	right ventricle
RVSP	right ventricle systolic pressure
*SiC*	control siRNA
*SiERK2*	*ERK2* siRNA
*TNF-α*	tumor necrosis factor-α
WT	wild type
VEGF	vascular endothelial growth factor
vWF	von Willebrand factor
